# Antibacterial Use of Macroalgae Compounds against Foodborne Pathogens

**DOI:** 10.3390/antibiotics9100712

**Published:** 2020-10-17

**Authors:** Aurora Silva, Sofia A. Silva, C. Lourenço-Lopes, C. Jimenez-Lopez, M. Carpena, P. Gullón, M. Fraga-Corral, V. F. Domingues, M. Fátima Barroso, J. Simal-Gandara, M. A. Prieto

**Affiliations:** 1 Nutrition and Bromatology Group, Department of Analytical and Food Chemistry, Faculty of Food Science and Technology, Ourense Campus, University of Vigo, E32004 Ourense, Spain; mass@isep.ipp.pt (A.S.); c.lopes@uvigo.es (C.L.-L.); cecilia.jimenez.lopez@uvigo.es (C.J.-L.); maria.carpena.rodriguez@uvigo.es (M.C.); patrigullon@gmail.com (P.G.); mfraga@uvigo.es (M.F.-C.); 2 REQUIMTE/LAQV, Instituto Superior de Engenharia do Porto, Instituto Politécnico do Porto, Rua Dr António Bernardino de Almeida 431, 4200-072 Porto, Portugal; vfd@isep.ipp.pt (V.F.D.); MFSBA@isep.ipp.pt (M.F.B.); 3 Departamento de Química, Universidade de Aveiro, 3810-168 Aveiro, Portugal; sofia.silva96@gmail.com; 4 Centro de Investigação de Montanha (CIMO), Instituto Politécnico de Bragança, Campus de Santa Apolonia, 5300-253 Bragança, Portugal

**Keywords:** macroalgae, microbial contaminants, antimicrobial compounds, food industry, safety and quality

## Abstract

The search for food resources is a constant in human history. Nowadays, the search for natural and safe food supplies is of foremost importance. Accordingly, there is a renewed interest in eco-friendly and natural products for substitution of synthetic additives. In addition, microbial contamination of food products during their obtaining and distribution processes is still a sanitary issue, and an important target for the food industry is to avoid food contamination and its related foodborne illnesses. These diseases are fundamentally caused by certain microorganisms listed in this review and classified according to their Gram negative or positive character. Algae have proven to possess high nutritional value and a wide variety of biological properties due to their content in active compounds. Among these capabilities, macroalgae are recognized for having antimicrobial properties. Thus, the present paper revises the actual knowledge of microbial contaminants in the food industry and proposes antimicrobial algal compounds against those pathogenic bacteria responsible for food contamination as valuable molecules for its growth inhibition. The capacity of algae extracts to inhibit some major food pathogen growth was assessed. Moreover, the main applications of these compounds in the food industry were discussed while considering their favorable effects in terms of food safety and quality control.

## 1. General View on Algae Compounds

Algae encompass a wide variety of photosynthetic living beings, usually aquatic, that comprise thousands of species. Even though the taxonomic classification of algae is a delicate subject, since each researcher classifies them according to different criteria, generally, marine algae are divided into microalgae and macroalgae or seaweed. Focusing on macroalgae, they are further classified into three main groups: green (Chlorophyta), brown (Phaeophyta) and red algae (Rhodophyta). Thousands of species are found in these three subgroups, many of them being of food interest [1]. In addition, they have been used in traditional medicine for many years against various diseases (tuberculosis, arthritis, colds and influenza) [2,3]. Seaweeds are part of the diet in many cultures, especially in Asia since they present a high nutritional value. Algae are a valuable source of protein (5–14%), fiber, lipids (<3%) and carbohydrates (13–19%), in addition to its content in micronutrients (such as salts, minerals and vitamins) necessary for the correct and healthy functioning of the organism [4,5]. Likewise, they are also rich in several secondary metabolites such as phenolic compounds, phycobiliproteins, carotenoids, alkaloids, terpenes, sulfated polysaccharides or phytosterols [6,7]. For these reasons, algae have been attracting curiosity also in the pharmaceutical industry for a few years, as they are very varied natural matrices that contain a great diversity of compounds with bioactive capabilities. Some of those beneficial biological functions to human health are antioxidant, anti-inflammatory, cardioprotective, antimicrobial, antifungal, antiviral and anticancer activities, among others [8]. Phytosterols extracted from various algae have demonstrated antimicrobial, analgesic and antioxidant functions, fucosterol being the predominant sterol in brown algae [4,9]. Other well-known molecules present in algae are sulfated polysaccharides which, in addition to presenting capabilities as gelling and thickening agents such as alginates and carrageenan, are associated with antioxidant, anticoagulant, antiviral, antitumor, anti-inflammatory and immunostimulant properties, such as the fucoidans present in the cell walls and the extracellular matrices of algae, especially the brown ones [10]. These reasons make them of great interest to the healthcare and food industries [11,12]. Phenolic compounds are associated with a strong antioxidant capacity, which is also associated with other beneficial properties such as anti-inflammatory, antimicrobial and anticancer. Phenolic compounds constitute a very heterogeneous group of molecules that can be classified in different ways. Notable among them are flavonoids, phenolic acids, lignans and tannins [13]. Within this last group are classified the so-called phlorotannins, molecules whose structure corresponds to polymers of phloroglucinol (1,3,5-trihydroxybenzene), which are present exclusively in algae, especially in brown. Phlorotannins can be deeper divided into four major groups (fuhalols and phlorethols, fucols, fucophloroethols and eckols), and are associated with powerful biological activities such as antioxidant, anti-inflammatory, antimicrobial, antifungal, antibiofilm or antifouling, hepatoprotective and antiviral [14,15]. Moreover, some fatty acids such as palmitic acid, linoleic acid, linolic acid, palmitoleic acid, eicosapentaenoic acid, stearic acid, or oleic acid have also demonstrated cardioprotective, antitumor and antimicrobial activities [16,17].

As described, the compounds contained in algae show numerous activities beneficial to health. Regarding the antimicrobial capacity of algae extracts, they have been successfully analyzed against various pathogens of great concern to human health. Some of the Gram positive bacteria studied are strains of *Bacillus subtilis*, *Bacillus cereus*, *Staphylococcus aureus, Enterococcus faecalis* or *Micrococcus luteus*, while the Gram negative bacteria analyzed include *Klebsiella pneumoniae*, *Serratia marcescens*, *Escherichia coli*, *Pseudomonas aeruginosa*, *Salmonella* Typhimirium or *Vibrio cholerae* [18]. The antimicrobial mechanisms of action associated with compounds extracted from marine algae are varied. These include the changes that occur in the permeability of the membranes thanks to the interaction with proteins and lipids present in them, or the ability to inhibit enzymes [17,19]. Some examples of specific compounds extracted from algae that have shown antimicrobial function are phlorotannins, laminarin, sargafuran, peyssonoic acid, bromophycolides, neurymenolides, acetylmajapolene and phycobiliproteins, phytol, fucosterol, neophytadiene, palmitic, palmitoleic and oleic acids [20,21].

The main current applications of algae include their use as fertilizers, in the production of biofuels, in the cosmetic industry and, of course, in food and agriculture [5,22]. An example of the application of compounds with antimicrobial capabilities obtained from macroalgae is their use in the livestock industry as food supplements, since it is a good strategy to reduce the use of antibiotics in animal feed. This substitution of antibiotics by natural functional molecules would help to combat the current growing resistance to antibiotics by pathogenic microorganisms [14,20]. Therefore, this abundant and ubiquitous source of bioactive compounds represented by algae could be used to obtain extracts rich in such antimicrobial compounds to be applied in the agri-food, cosmetic or pharmaceutical industries.

## 2. Microbial Contamination in Food Industry

Feeding habits are undergoing drastic changes since consumers are increasingly searching for natural food ingredients. Synthetic additives are generating rejection due to the harmful health effects associated with them, such as certain neurological or immune conditions (hyperactivity, allergies, etc.). This situation has triggered the research for new natural sources of bioactive compounds that can exert actions that improve or extend the shelf life of products, such as preservative function, among others, which has become a trending topic for the food industry [21].

On the other hand, the appearance of microbial contamination in food products during their processing, storage, distribution and consumption is a sanitary problem that the mentioned industry also must face. This contamination may be favored by intrinsic factors in food, such as bacterial load or its water content, or by external factors that condition the stability of the food, such as the presence of oxygen, humidity or temperature of the environment [23]. To avoid this contamination that can lead to foodborne illnesses, different food safety control strategies are carried out such as hazard analysis and critical control point (HACCP), sanitation standard operating procedure (SSOP) and good manufacturing practices (GMP), the first being the most widely used globally. However, these strategies also have drawbacks such as the detection and determination of the critical control points. In this regard, novel techniques that allow contamination control while guaranteeing food safety are required. These controls are necessary to prevent specific contaminations that can lead to cross-contamination throughout the food production and consumption chain [24].

Some typical food contaminants are *Pseudomonas* sp., *Listeria monocytogenes*, *Salmonella* sp., *Shigella flexneri*, *V. cholerae*, *E. coli*, *Bacillus* sp. and *Campylobacter jejuni.* Additionally, many of the pathogens responsible for contaminations that lead to foodborne illnesses can form biofilms, making them even more harmful and difficult to control. There are various techniques to sterilize food so that microbial contamination can be avoided, such as the use of ozone, electrical pulses, ultrasounds, microwaves, radiation or thermal processes [25]. However, as aforementioned, algae are rich in bioactive compounds such as alkaloids, terpenoids, polysaccharides, pigments, cyclic peptides, phenols, lipid, and vitamins [20], many of which show antimicrobial and anti-biofilm properties that have been successfully tested against some of the common food pathogens. For this reason, compounds obtained from marine algae that show antimicrobial capabilities could be used alternatively, since their use would be doubly advantageous, both to mitigate microbial contamination in food and to replace certain synthetic additives, increasing the attractiveness of the products to consumers, their value and being healthier since synthetic preservatives could be replaced [20,26].

This work collects information about natural compounds extracted from marine algae that show antimicrobial capabilities against pathogenic bacteria commonly responsible for food contamination that can cause foodborne illnesses. Furthermore, potential applications of these bioactive compounds in the food industry are described, discussing their compliance with quality and safety controls, and finishing with some future possible approaches.

## 3. Antibacterial Potential of Bioactive Compounds from Macroalgae against Pathogens and Spoilage Microorganisms

### 3.1. Evaluation Criteria

Agar diffusion methods are frequently used to evaluate the antimicrobial capacity of new compounds. In this microbiological technique, agar plates are inoculated with a standardized inoculum of the tested microorganism. Then, filter paper discs containing the tested compound at the desired concentration are placed on the agar surface. Generally, the antimicrobial agent diffuses into the agar and inhibits germination and growth of the tested microorganism and then the diameters of inhibition growth zones are measured [27]. Nevertheless, there is no standard way to carry out a qualitative evaluation. Furthermore, different authors have used different scales [28,29,30,31]. Therefore, to standardize the classification, this review will adopt the classification proposed by the Clinical and Laboratory Standards Institute (CLSI). Depending on the measured diameter of the inhibition growth zone, the bacteria can be categorized as susceptible (≥20 mm), intermediate (I, 15–19 mm) or resistant (R, ≤14 mm) [32].

Another standard way to evaluate the antimicrobial susceptibility of extracts or purified compounds is the broth dilution test. In this experimental approach, the test substance is diluted in a broth or saline solution and added to the broth containing antimicrobial agent, the microorganism in the mixture can grow for 16 to 24 h. The result is normally quantitative and expressed as minimal inhibition concentration [33,34].

### 3.2. Gram Negative

#### 3.2.1. Enterobacteriaceae

Members of the *Enterobacteriaceae* family possess Gram-negative stain properties and they are facultative anaerobes, fermenting sugars to produce lactic acid and other various end products. The family *Enterobacteriaceae* includes many bacteria that are found in the human or animal intestinal tract, including human pathogens. *Enterobacteriaceae* are useful indicators of hygiene and post-processing contamination of heat-processed foods.

##### *Escherichia coli* 

*Escherichia coli* is an oxidase-negative rod-shaped bacterium. This bacterium grows preferably at a temperature of 37 °C and can either be nonmotile or motile, presenting in its constitution peritrichous flagella. Even though *E. coli* can be a harmless resident in the gastrointestinal tract, it also has the pathogenic capacity to cause significant diarrheal and extraintestinal diseases. Pathogenic variants of *E. coli* cause significant morbidity and mortality worldwide [35]. One of the most important sources of foodborne diseases worldwide is caused by the Shiga-type toxins produced by the *E. coli* strains serotype O157:H7. These strains, in particular, started to be considered as a significant risk to public health in the 1980s, when they were associated with outbreaks of gastrointestinal symptoms and hemolytic uremic syndrome [36]. In 2018, in the European Union, a total of 2.28 citizens per 100,000 habitants were contaminated with *E. coli* related food poisoning [37]. In the years between 2003 and 2012, the United States recorded 390 *E. coli* O157 type outbreaks, with 65% of them being food-related [38].

Most of the *E. coli* associated foodborne outbreak cases over the past decade have been attributed to the consumption of uncooked foods (raw source or during the preparation process) contaminated by pathogenic *E. coli*. Furthermore, *E. coli* did not only cause huge economic losses but also impacted human health and even caused death [38,39]. *E. coli* is widely selected as a target microorganism to assess the antimicrobial activity of compounds and crude extracts from numerous macroalgae species (Table 1). In this review, it was found that the most tested *E. coli* strain was the American Type Culture Collection (ATCC) 25922 [40,41,42,43,44,45,46,47,48,49,50,51,52,53]. Nevertheless, methanolic extracts of the brown algae *Scytosiphon lomentaria* and *Padina pavonica* inhibited the growth (diameter > 15 mm) of the *E. coli* erotype O157:H7 [54].

The most studied species are brown algae, followed by the red and finally the green algae. As it can be seen in Table 1, brown algae [28,50,54,55,56,57,58,59,60] promoted inhibition growth zones larger than 15 mm diameter, allowing the antibacterial activity categorization of intermediate or susceptible. Minimal inhibition concentration (MIC) values ranging from 7.5 mg/L to 0.16 mg/L were achieved when an acetone-based crude extract of *Cutleria multifida* was used [42]. In the red algae group, the *Gracilaria multipartite* [56,58], *Corallina officinalis* [50], *G. corticata* [61] and *Rhodomela confervoides* [60] were the algae that presented antibacterial activity between intermediate to susceptible against *E. coli*. Moreover, the alga *Laurencia papillosa* contains several bioactive molecules such as pigments (chorophyll, carotenoids, phycocyanine, phycoerythrine), proteins, carbohydrates, phenolics, flavonoids, tannin, fatty acids in its composition, presenting the lowest MIC (0.2 mg/mL) [62].

In the Chlorophyta algal group, the *U. flexuosa* [66], *C. vagabunda* [67], *U. lactuca* [56,79], *G. multipartite*, *C. glomerata* [50]; *Hypnea valentiae* [56], *Stigeoclonium* sp., *Ulothrix* sp., *Nitzschia* sp. [72], *E. prolifera*, *U. reticulata* [81] and *U. rigida* [45] showed the most promising antibacterial capacity since they were able to inhibit *E. coli* growth in the intermediate or susceptible category. *U. flexuosa* [66] produced diameters of the inhibition growth zone larger than 20 mm and MICs of 1.18 and 0.93 mg/mL for the methanolic and ethyl acetate extracts, respectively. Studies made on *S. polycystum* and *P. australis* by Hauck [48] showed that crude extracts of algae collected from different locations gave slightly different MICs. In addition, some research made using the red algae *G. multipartite* showcased that there was a connection between the season when the specimen was collected and its antimicrobial effect against *E. coli* [56]. This effect was further proven by an antibiogram made using the algae *U. rigida* [80]. The main technique used to obtain bioactive compounds from algae is maceration using different solvents, even though chemicals such as chloroform are still used, the majority of studies use methanol, ethanol and water, as shown in Table 1. The solvent effect of *Nitzschia* sp. and *Stigeoclonium* sp. ethanolic extracts on *E. coli* growth leads to a higher diameter of the inhibition growth zone than methanolic ones, but no significant difference was found when *Ulothrix* sp. extracts were used. The findings reported on the antibacterial activity from marine algae extracts from the Red Sea extracted with different solvents implied that petroleum ether, diethyl and ethyl acetate were the best extractive solvents for extracting bioactive compounds (catechin, phlorotannins flavonols, glycosides flavonol and phenolic compounds), followed by methanol [72]. On the other hand, chloroform-based crude extracts of *U. lactuca* presented a higher antibacterial activity than diethyl ether and methanol ones. However, in the same study, *D. dichotoma* methanolic extract displayed better antimicrobial activity [79]. This implies that solvent selection is critical and should be optimized for each alga.

##### *Enterobacter* spp.

*Enterobacter* spp. are foodborne pathogens responsible for opportunistic infections. The genus *Enterobacter* consist of 12 species, including *E. cloacae* and *E. aerogenes*—the most commonly isolated species that cause human infections [82]. Studies were conducted to examine the effects of the temperature and relative humidity on the survival of *E. aerogenes* on stainless steel, polyvinyl chloride and ceramic tile. Although surface type had little effect on bacteria survival, the temperature had a clear effect on the *E. aerogenes* survival. In this case, bacterial growth presented its maximum at 7 °C and 15 °C and 50% relative humidity on all surfaces [83]. Moreover, in 46 pepper samples from Vietnam, the *E. cloacae* were isolated in 30 samples (65.2%) [84].

Concerning the antagonistic activity of algae extracts against *Enterobacter* spp., the literature is scarce as shown in Table 2. The most common alga group tested is Phaeophytha phylum. However, all extracts tested were classified as resistant or present with no antimicrobial activity against *Enterobacter* spp. On the other hand, the outcome obtained with purified ulvan, a cell wall polysaccharide formed by bioactive molecules such as rhamnose, galactose, xylose, mannose and glucose, uronic acid and sulphate groups, from the green alga *U. reticulata* excels with a report of a diameter of the inhibition growth zone of 20 mm [78]. It is also interesting to bring to light that in the range of algae shown in Table 2, *U. reticulata* is not only the one that shows the best results but also is the only green algae tested.

##### *Shigella flexneri* 

*Shigella* spp. is an etiological agent of bacillary dysentery or shigellosis. This disease is marked by fever, violent abdominal cramps and rectal urgencies [85]. *Shigella* spp. causes annually approximately 500,000 illnesses, 6000 hospitalizations and 40 deaths in the United States [85]. *S. dysenteria* is usually present in the epidemic and outbreak forms of dysentery disease. *S. flexneri* and *S. sonnei* are mainly responsible for endemic shigellosis disease in developing and developed nations, respectively. While *S. boydii* is restricted to India and neighboring countries, the *S. flexneri* was found to be the most commonly isolated species (68%) in a multi-centric study from six Asian countries including China, Vietnam, Thailand, Bangladesh, Pakistan and Indonesia [86].

Unfortunately, *Shigella* spp. have presented resistance to most used antibiotics, which is a serious concern to health [87]. Usually, food is contaminated through *Shigella* spp. by an infected food handler who practices poor personal hygiene. Moreover, *Shigella* spp. is acid resistant, salt-tolerant and can survive at infective levels in many types of foods such as fruits and vegetables, low pH foods, prepared foods and foods held in a modified atmosphere or vacuum packaging [85]. Studies involving brown algal extracts against *S. flexneri* were conducted [76,88] and the majority of the tested algae species belonged to the *Sargassum taxa,* as shown in Table 2. From observing Table 2, we can notice that all the antimicrobial test results were attained by Kirby–Bauer test and presented no quantitative results. Nevertheless, the capacity of different solvents to extract bioactive compounds (indoles, terpenes, acetogenins, phenols, fatty acids and volatile halogenated hydrocarbons, and also high percentage of octadecadienoic acid, eicosane, dotriacontane, tritetracontane, docosane, octatriacontyl, heptacosane and tetracosane) [88] able to promote the inhibition growth of *S. flexneri* was evaluated [88] and the highest inhibition was obtained with *S. vulgare* methanol (MeOH) and diethyl (DiEt) crude extracts; *Sargassum fusiforme* DiEt > chloroform (CHCL_3_) > ethanol (EtOH); *P. pavonica* EtOH > CHCL_3_ > DiEt and MeOH; *Ceramium rubrum* EtOH > CHCL_3_ DiEt and MeOH.

##### *Proteus* spp.

*Proteus* spp. microorganisms are widely distributed in the natural environment, including water, soil and manure. Due to their proteolytic activity, the ability to hydrolyze urea to ammonia and carbon dioxide, as well as the oxidative deamination of amino acids, these bacteria are involved in the decomposition of organic matter of animal origin [89]. Currently, the genus *Proteus* is divided into *Proteus mirabilis*, *P. vulgaris*, *P. penneri*, *P. hauseri*. These bacteria are known to be human opportunistic pathogens, isolated from urine, wounds and other clinical sources [90]. Some *P. mirabilis* strains were found to be associated with food poisoning outbreaks [91]. This bacterium has been found in several food animals such as poultry, especially chickens [92,93]. Ten prepared vegetable salads obtained at various sales points in Ekpoma, Nigeria were investigated for the isolation of these bacteria. The results demonstrate the presence of *Proteus* sp. in 20% of the samples [94]. New multiple resistant forms of this bacteria have been discovered. Therefore, it may become a major food issue in the future [95]. *P. vulgaris* exhibited the highest resistance to the antibiotics, with a value of six out of eleven antibiotics (54.5%) [96].

In the last decade, most of the studies on algal antimicrobial compounds include (Table 2) *Proteus* spp., namely the strains of *P. vulgaris* [97,98] or *P. mirabilis* [29,62,99,100]. The brown algae *T. conoides*, *P. gymnospora* and *Sargassum tenerrimum* were extracted with various solvents: methanol, acetone, petroleum ether, ethanol, ethyl acetate, chloroform and diethyl ether. The obtained crude extracts were tested against *Proteus* sp. The most favorable inhibition results were achieved with *T. conoides* acetone extract, methanolic- and chloroform-based extracts of *P. gymnospora* and petroleum ether extract of *S. tenerrimum* [101]. Purified extracts from several brown algae, rich in phlorotannins, evidenced antimicrobial activity against several pathogens including *P. mirabilis* [102]. A MIC of 0.65 mg/mL was obtained with the n-butanol fraction of *Liagora hawaiiana* extract [97]. Epitaondiol, epitaondiol diacetate, epitaondiol monoacetate, stypotriol triacetate, 14-ketostypodiol diacetate and stypodiol were successfully isolated from the dichloromethane (DCM) crude extract of brown alga *Stypopodium flabelliforme* and these compounds demonstrated antimicrobial activity against *P. mirabilis* (MICs below 1 mg/mL) [103].

##### *Salmonella* spp.

Salmonellae are flagellated organisms that are ubiquitously distributed and cause salmonellosis. Non-typhoidal salmonellosis is a worldwide disease of humans and animals. Animals are the main reservoir, and the disease is usually foodborne, although it can be spread from person to person [104]. Around the world, several outbreaks of food poisoning related to *Salmonella* are documented, e.g., an outbreak of infection with non-typhoidal *Salmonella* affecting 396 patients in the community was associated with frozen pies supplied for cooking by the consumer in a microwave oven [105]. During a prevalence study made in Asia involving 807 meat samples, 159 (19.7%) were positive for *Salmonella*. Pork had the highest prevalence (37.3%, 107/287) of *Salmonella* contamination, followed by beef (16.1%, 26/161), mutton (10.9%, 10/92), dumplings (6.6%, 14/212) and smoked pork (3.6%, 2/55) [106]. A report focusing on multidrug-resistant Salmonella in the United States in years 2018–2019 linked 255 infections to contaminated beef and soft cheese [107]. From a work focused on chickens, a total of 384 caecal content samples were collected for microbiological examination and the overall prevalence of *Salmonella* was 14.6% [108], showing the ubiquity of this bacteria.

Overall, the most studied salmonella species in antimicrobial tests performed with alga extracts is *Salmonella* Typhi. Studies made with these bacteria species allow the classification of the antimicrobial activity of intermediate to susceptible (diameter of the inhibition growth zone higher than 15 mm) from the Phaeophyta phylum: *D. membranacea* [28], *P. gymnospora* [47], *D. dichotoma* var intricate (B), *D. indica* (B) [57], *S. lomentaria* (B) [54], and *Ecklonia cava* [109]. Among the Chlorophytes and Rhodophytes, *U. rigida* and *C. officinalis* [50] also present antagonistic effects to S. Typhi. Alga extracts were also tested against additional *Salmonella* spp.: *S.* Enteridis, *S.* Choleraesuis and *S.* Gallinarum. Although several extracts presented no activity in Table 2, the ethyl acetate fraction extract of *E. cava* presented a MIC of 0.25 mg/mL [109].

#### 3.2.2. Pseudomonadaceae: *Pseudomonas aeruginosa*

*Pseudomonas aeruginosa* is an encapsulated and rod-shaped bacterium that is citrate, catalase and oxidase positive. *P. aeruginosa* can be found in water, soil, skin flora and most man-made environments throughout the world. This bacterial pathogen can cause broad human illnesses comprising osteomyelitis, endocarditis, pneumonia, meningitis, septicemia, urinary tract and gastrointestinal infections [110]. The Antibiotic Resistance Threats in the United States (2019) predict a total of 32,600 cases and 2700 fatalities, due to multidrug-resistant *Pseudomonas aeruginosa* [111]. Several studies investigated the level of *P. aeruginosa* contamination of commercially supplied packaged water around the world [112]. A study promoted by Sotler and collaborators [110] indicated the presence of *P. aeruginosa* in 33 of the 80 tested sachet water samples. The presence of this bacterium was reported in seven sachet water samples supplied in India [113]. In Abuja, Nigeria (2014), *P. aeruginosa* was isolated in 9.36% out of 100 brands of packaged water samples [114]. A similar investigation involving 120 sachet water samples collected from Owerri, Imo State, Nigeria (2011) revealed *P. aeruginosa* in 12% of the samples [115]. Furthermore, *P. aeruginosa* was also isolated from 8 of 38 routine post-pasteurization milk samples. In this strange case, all the pre-pasteurization milk samples were sterile. The *P*. *aeruginosa* contamination occurred during the water intake pipes in the milk bank and from cold water immersing milk bottles immediately after pasteurization [116].

*P. aeruginosa* growth was suppressed by an intermediate grade of *C. barbata* [50], *C. tamariscifolia* [60] and *P. pavonica* [60,88] (Phaeophyta), *C. glomerata* [50] (Chlorophyta) and *L. papillosa* [62], *R. confervoides* [60] and *C. rubrum* [88] (Rhodophyta). The extraction solvents most used are methanol and ethanol, as shown in Table 2. After a phytochemical screening, the alga species *C. rubrum*, *S. vulgare*, *S. fusiforme* and *P.pavonia* showed the presence of indoles, terpenes, acetogenins, phenols, fatty acids and volatile halogenated hydrocarbon. These alga were extracted and the solvent effect was investigated with diethyl ether, methanol, ethanol and chloroform, reporting the following results: *S. vulgare* presented results only with diethyl ether, *S. fusiforme* showed activity with diethyl ether > methanol > ethanol > chloroform, *P. pavonica* showed activity with chloroform > diethyl ether and ethanol > methanol. *C. rubrum* showed activity with diethyl ether > ethanol > methanol and chloroform [88]. In another study focused on *P. australis* and *S. polycystum* from two different locations, best inhibition results were achieved with methanol and dichloromethane for *P. australis* and hexane for *S. polycystum*. Algae from different locations gave slightly different MICs [101].

### 3.3. Gram Positive

#### 3.3.1. *Staphylococcus aureus*

*Staphylococcus aureus* is a facultative anaerobic coccus, which is non-motile and catalase and coagulase positive. Among the predominant foodborne bacteria described as human pathogens, *S. aureus* is the foremost cause of gastroenteritis [122], but contamination can be readily avoided by heat treatment of food. Researchers assessed the prevalence of *S. aureus* and its enterotoxins in raw pork and smoked hams produced from this meat [123]. In total, the pathogen was found in 25.9% of the 135 samples by culture techniques and *S. aureus* genes were detected by polymerase chain reaction (PCR) in 51.1% of the examined samples. From 11.1% of the ready-for-sale smoked hams, *S. aureus* was isolated and 35.6% of this product reacted positively in the PCR. Therefore, concerning the consumers’ health, these results have to be critically evaluated [123]. Although *S. aureus* can be easily found on the skin and hair of warm-blooded animals, up to 30–50% of the human population are carriers of these bacteria [122]. This bacterium is notorious for its resistance to multiple antibiotics [124].

Concerning the antibacterial activity from bioactive compounds from macroalgae on *S. aureus*, several studies have been performed within Chlorophyta, Rhodophyta and Phaeophyta phylum.

Usually, different extraction solvents are used to obtain bioactive compounds (alkaloids, polyketides, cyclic peptide, polysaccharide, phlorotannins, diterpenoids, sterols, quinones, lipids and glycerols) [29]: methanol (most used), ethanol, acetone, dichloromethane, hexane, diethyl ether, ethyl acetate, dimethilsulfoxide (DMSO), chloroform and solvent mixtures. The strain most evaluated was ATCC 25923 but also activity against methicillin-resistant S. aureus (MRSA) was tested, as shown in Table 3. Acetone extracts of *L. obtusa*, *C. elongatum* and *C. multifida* obtained the lowest MIC at 0.625 µg/mL [125]. Among Chlorophyta, the genus *Ulva* spp. was reported in most of the studies against *S. aureus* [45,50,66,80,125,126,127,128]. Some studies compared solvent extraction performance for extraction of bioactive compounds: for *C. socialis* (MeOH > acetone) [118], *U. flexuosa* (ethylacetate > MeOH) and for *U. rigida* (ethanol > Hex> CHCl_3_). Regarding Rhodophyta, the genus with the most described studies is *Laurencia* spp. [52,62,77,125,127]. Relating to Phaeophytha phylum, *Sargassum* spp. was the most studied genus [41,57,73,76,99,118,129,130]. Comparing solvent performance for bioactive compounds extraction, the following results were obtained: ethanol or acetone > crude extract or MeOH/DCM for *D. membranacea* [28], EtOAc > EtOH > hexane, DCM, n-BuOH, water (H_2_O) for *E. cava* [109]; EtOAc > DCM, BuOH> hexane > MeOH for *E. bicyclis* [121]; H_2_O > MeOH, acetone or ethyl acetate for *F. serratus* [131]; ethanol > hexane > H_2_O for *H. elongata* [21]; ethylacetate > MeOH for *P. antillarum* and *P. boergeseni* [66]; MeOH > acetone for *S. platycarpum* and for *S. latifolium* [118]. It can be concluded that there is not a common solvent to extract compounds with antibacterial activity against *S. aureus.*

#### 3.3.2. *Listeria monocytogenes*

*Listeria monocytogenes* is a non-spore-forming, facultative anaerobic foodborne pathogen that can reproduce at refrigeration temperatures as well as at acidic pH and high salt concentrations and shows high resistance to disinfectants. In the food industry, this pathogen can colonize the environment, equipment and utensils and form biofilms, where it can remain for several months or even years causing cross-contamination [133]. Listeriosis is a serious infection usually caused by eating food contaminated with the bacterium *L. monocytogenes*. In major cases of young people and adults, it provokes a central nervous system infection and/or generalized infection. However, it is particularly dangerous in pregnancy, due to the ability of this bacterium to cause intrauterine infection and severe systemic infections in the unborn or newly delivered infant. Listeriosis can also arise in any gestation phase and affects children under one month old [134].

The prevalence of *Listeria* sp. and specifically *L. monocytogenes* in 650 ready-to-eat products (RTE) and 263 ingredients of salads and desserts was evaluated in Poland [135]. Unfortunately, 18% of the samples were contaminated with *Listeria* sp. and 13.5% with *L. monocytogenes*. [135]. Moreover, 124 RTE samples composed of ground beef and chicken meat were also assessed and 81.5% of the samples presented contamination with *Listeria* sp., 35.5% with *Listeria innocua* and 26.6% with *L. monocytogenes.* In the years of 1998–2014, a total 58 outbreaks of listeriosis were reported in the US, 30% of those cases were associated with soft cheese [136].

Brown alga extracts obtained with different solvents were tested against *Listeria* sp. growth. Experiments made with *D. membranacea* (extracted in H_2_O) [59] and *C. glomerata* (extracted in EtOH) [50] led to inhibition halos of intermediate diameter (Table 4). On the other hand, the brown alga species *Laminaria digitata, Laminaria saccharina, H. elongata* and the red algae *Palmaria palmata,* a seaweed rich in tannins, phenolics and flavonoids, were able to suppress the *Listeria* sp. growth ranging from very strong to moderate intensity [137].

#### 3.3.3. *Enterococus* spp.

*Enterococcus* spp. can be found in several reservoirs, namely soil, water, vegetable products, meats, fermented and cooked meat and dairy products. Furthermore, these microorganisms are present as common microbiota in the intestine of humans, mammals and other animal gastrointestinal tracts. *Enterococcus* spp. is a facultative anaerobe bacterium proficient to subsist at high temperatures and in a broad range of pH conditions [138]. However, due to its adaptability, *Enterococcus* can endure several niches, serving as a parameter in sanitary quality of food [139].

Epidemiology studies referred to *Enterococcus* spp. as a human pathogen liable for 5% to 10% of endocarditis infections [140], in addition to being responsible for other illnesses such as urinary tract, central nervous system and pelvic infections. *Enterococcus* resistant to the glycopeptide antibiotics, vancomycin and teicoplanin, are also emerging [111,141]. Algae extracts were tested mainly against two different *Enterococcus* species, *E. faecalis* and *E. faecium.* The growth of *E. faecium* was successfully constrained by the crude extract of *D. membranacea* and by the ethanolic and acetone crude fractions containing the following bioactive molecules: catechin, polyphenols, tannin and phlorotannins [28], whereas the chloroform fraction of the green alga *U. rigida* crude extract (rich in phenolics, diterpenoids, terpenes, sterols, fatty acids), proved its efficiency against the *E. faecalis* [45]. Some authors have also studied the seasonal influence of the *U. rigida* performance as an antimicrobial agent. They showed that the algae harvesting season has an effect on the performance of the extract [142].

#### 3.3.4. *Bacillus cereus*

*Bacillus cereus* is a facultative aerobic spore-forming bacterium able to thrive in temperatures ranging from 7 °C to 30 °C [143]. This bacterium is a well-known foodborne pathogen, frequently widespread in soil and growing plants, nevertheless it is also able to grow in the intestinal tract of insects and mammals. From these habitats, it is easily spread to foods, where it may cause an emetic or a diarrheal type of food-associated illness that is becoming progressively more prevalent in the food industry [144,145]. An extensive study on the prevalence of *B. cereus* isolated from dairy products in China was performed [146]. *B. cereus* strains were isolated from 500 dairy product samples and a contamination rate of 11% was achieved. Raw milk presented the highest *B. cereus* contamination rate (26%) followed by pasteurized milk samples (12%), cheese samples (10%), ultra-pasteurized milk samples (8%) and powder infant formula samples (7%) [146]. In another survey, approximately three hundred *B. cereus* strains were isolated from vegetables in different cities in China and were detected in 50% of the 419 samples [147].

Even though *B. cereus* spreads by spore formation, there is not enough evidence of the relationship between toxin production and biofilm formation. Moreover, there is also scarce evidence of the role of sporulation in the toxin production of *B. cereus*. As a foodborne pathogen, toxin production is important, and any sporulation and biofilm formation are expected to worsen the risk of food poisoning [145]. In the pursuit of alga extracts with an antagonistic effect on *B. cereus* growth, a large number of algae was tested by the agar diffusion method, but most were included in the resistant category, as shown in Table 4. Nevertheless, cholesterol and oxygenated cholesterol derivatives, brominated indoles 1–3 along with sesquiterpene, debilone, and mixture of fatty acid esters detected [77] in *P. coriaceum, C. antennina* and *Codium cylindricum* acetone extract [29] and in *L. complanata* (R) methanolic extract, presented encouraging results with the diameter of the inhibition growth zone higher than 15 mm. The green seaweed *E. linza* was extracted to obtain essential oils and tested against two different *B. cereus* strains: ATCC 10876 and 13061. However, the MIC results were quite different, inferring that the strain of the microbe tested played an important role in the final experiment outcome [148]. It was also noticed that the inhibition capacity of the extracted compounds from *P. coriaceum* collected in different locations also corresponded to slightly different results [29].

#### 3.3.5. *Streptococcus* spp.

The genus *Streptococcus* consists of 104 recognized species, both commensal and pathogenic. Major pathogenic species for human and animals are *S. pneumoniae* (middle ear infections, meningitis and pneumonia in children and pneumonia with sepsis in adults or immunocompromised persons), *S. pyogenes* (scarlet fever, rheumatic fever), *S. agalactiae* (neonatal pneumonia in human, mastitis in animals), *S. dysgalactiae* subsp. dysgalactiae [149] (mastitis), *S. dysgalactiae* subsp. *equisimilis* (strangles in horses, joint ill, mastitis in animals), *S. gordonii* (infective endocarditis in human), *S. parasanguinis* (infective endocarditis in human), *S. sanguinis* (infective endocarditis in human) and *S. mutans* (dental caries). An epidemiological investigation performed by Yamaguchi et al., [150] revealed the presence of *S. dysgalactiae* in a broccoli salad. Unfortunately, this contaminated food provoked a foodborne outbreak and 140 patients presented primary symptoms of sore throat and fever [150]. Bovine mastitis is the most frequent disease worldwide in dairy herds, causing high economic losses for producers and the industry, as well as having implications for public health due to the zoonotic potential of some agents involved in its etiology and the increased risk of antimicrobial residues in milk and its derivatives [151].

The prevalence of *S. agalactiae* in 306 dairy herds from Campo das Vertentes region, located in the south of Minas Gerais state, Brazil, was evaluated. The study involved approximately 3.4 × 10^4^ dairy cows and covered an area of approximately 1.2 × 10^4^ km^2^ and indicated that 67% of the samples were contaminated with *S. agalactiae* [151]. *S. agalactiae* was also found in bulk milk from Prince Edward Island dairy farms [152]. Furthermore, a high prevalence of *S. infantarius* was found in fermented milk of East and West Africa [153] The most relevant results related with the antimicrobial capacity of algae against *Streptococcus* spp. are summarized in Table 4. Among the algae tested against *S. pneumoniae,* the best inhibition results were obtained with *S. incisifolium, Sargassum* sp. [76]—an algae containing sterol and terpens—and *L. complanata* extracts [77]. The extracts of *G. rugosa* and *L. hawaiiana* were active against *S. mutans* [97]. The extract of *G. rugosa* also negatively affected the growth of *S. pyogenes.* Several seaweeds extracts (*T. conoides, P. gymnospora* and *S. tenerrimum)* obtained with different solvents were tested against *Streptococcus* sp. and the best inhibition results were obtained with acetone, ethyl acetate and methanol, respectively [101]. *Laminaria japonica* ethanolic extracts were used to restrain the growth of oral related bacteria (*S. gordonii, S. mitis, S. mutans, S. oralis, S. sanguinis and S. sobrinus*). MICs were between 60 to 250 µg/mL, indicating that it is reasonable to assume future applications in dental care products.

## 4. Incorporation to the Food Industry

**Figure 1:**
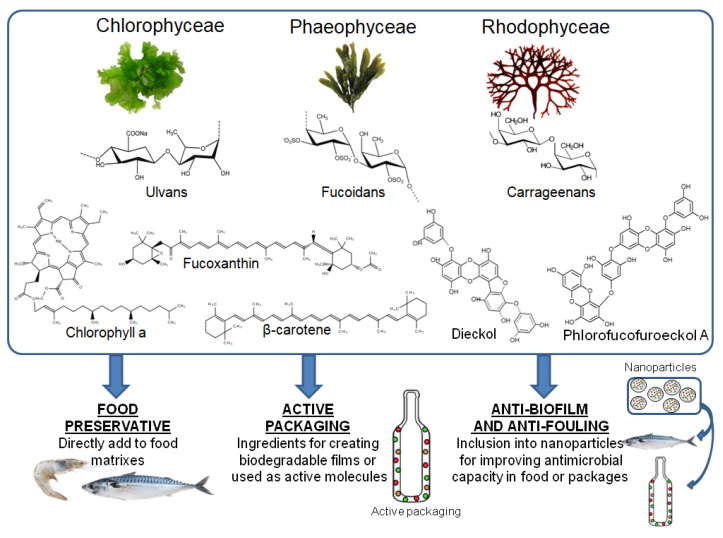
Food applications of macroalgae extracts. Green (Chlorophyceae), brown (Phaeophyceae) and red (Rhodophyceae) macroalgae biomolecules can be used for: (a) their direct application into food products, (b) the development of biodegradable packages and/or for their incorporation as active ingredients into films (active packaging), (c) for their inclusion into encapsulation systems that can be further used for their application in food matrixes or active packaging.

Nowadays, many molecules have been recognized as permitted additives. They mostly avoid food spoilage induced by oxidant reactions, microbial growth and/or browning processes. Some of the permitted additives with antioxidant capacity are ascorbates, tocopherols, gallates, butylates, lactates, citrates or phosphates, among many others [158]. Those additives considered to exert microbial inhibition growth include acetic, malic, lactic, benzoic, sorbic and propionic acids and some of their salts, as well as parabens. Sulfites are the most used additives for avoiding food browning caused by any chemical or enzymatic reactions. Nevertheless, several natural compounds represent a current alternative to the use of chemical anti-browning ingredients, such as erythorbic acid, cysteine, 4-hexylresorcinol and some phenolic acids [159]. In fact, this is the current trend in the food industry—replacement of chemically synthesized compounds with natural ones. Consumers’ claims have prompted this shift due to the side effects related to the consumption of chemically synthesized molecules. Currently, macroalgae represent a promising source of natural molecules with a variety of recognized bioactivities such as antioxidant and anti-microbial, among many others. The high content in polyphenols, such as the phlorotannins that can reach up to 15% of the dry matter, or pigments, such as carotenoids and chlorophylls, are mainly responsible for their antioxidant activities [160]. It is also known that the diversity of polysaccharides present in macroalgae, carrageenans and agar from red algae, fucoidans from brown and ulvans from green ones, possess antibacterial capacities [161]. Apart from their richness in biomolecules and bioactivities, macroalgae have been demonstrated to represent a cheap, available and eco-friendly source of compounds, which results in being very interesting for the food industry [160,162,163]. Currently, algae extracts have been evaluated as food additives for food preservation, as ingredients for creating biodegradable films or core ingredients in active packaging with several functions such as anti-biofilm or anti-fouling agents, as shown in Figure 1.

### 4.1. Food Preservative

The use of algal extracts by the food industry for improving the conservation of different food matrixes has been used for more than two centuries [164]. For instance, polyphenols and polysaccharides from *Porphyra yezoensis* extended the shelf-life of *Litopenaeus vannamei* when refrigerated. The efficiency of conservation was evaluated through several parameters considered for the determination of food spoilage: colony-forming units (CFU), pH, total volatile basic nitrogen (TVB-N) or thiobarbituric acid (TBA), among others. Results demonstrated that the application of these algal compounds were able to inhibit the shrimp spoilage and prolong its shelf-life for a further 3–4 days [164]. Similarly, extracts from *G. verrucosa* also permitted to extend the sensory score of Indian mackerel (*Rastrelliger kanagurta*) by 4 days when compared to the traditional ice bath storage. As in the previous research, different spoilage parameters such as TVB-N, pH, bioamines concentration and microbial proliferation were studied. The presence of the polyphenols from the alga significantly inhibited the microbial growth, delayed the increment of spoilage markers and prevented the formation of biogenic amines [165]. Very similar results were observed when using *C. compressa* extracts for conserving chilled *Trachurus trachurus.* The presence of the alga inhibited the microbial activity and the chemical spoilage markers while reducing those related to lipid hydrolysis and oxidation [166]. Other work also stated the preservative ability of polyphenols obtained from *Fucus spiralis* and *U. lactuca* when incorporated as a canning medium for *Scomber colias*. The presence of the algae inhibited the breakdown of free fatty acids and the increment in the peroxide value, especially for samples treated with *F. spiralis* [167]. Therefore, the incorporation of algae compounds into fish or shellfish matrixes provides extended storage periods while reducing the microorganism growth rate, which ultimately improves food safety.

### 4.2. Active Packaging

The application of active packaging is an innovative solution for improving the storage time of food products. This approach consists of activating a film material by the inclusion of molecules with recognized bioactivities. Usually, the two main issues concerning any food matrix are the oxidation reactions and the microbial spoilage, thus the main bioactivities expected to accomplish by active packaging are antioxidant and antimicrobial properties. Macroalgae have served as a source of natural biomolecules with antioxidant and antimicrobial capacities to create active packaging systems but also as a source of biomaterials for creating biodegradable films.

The extraction of algae molecules has been widely optimized for their further incorporation into films. For instance, differential heat treatments of *Mastocarpus stellatus* with/without enzymatic hydrolysis permitted to obtain several extracts with strong antioxidant activity due to the presence of polyphenols or higher contents of κ/ι-hybrid carrageenan or protein. The mixture of three of these extracts, using glycerol as plasticizer, provided a film characterized by the presence of proteins and sulfated compounds that presented high water resistance and appropriate mechanical properties. Additionally, this film possessed effective antioxidant activity, quantified as 70.60 ± 0.47 mg of vitamin C equivalents per g (ABTS), 1.16 ± 0.04 μmol of Fe^2+^ per g (FRAP), 41.32 ± 3.19 mg gallic acid equivalent per g of sample (Folin-reactive substances) [168,169].

A polylactic acid-based biodegradable film was incorporated with 8% of *F. spiralis* extract and 1% of sorbic acid. Its protective effect was evaluated during the refrigerated storage of megrim (*Lepidorhombus whiffiagonis*). For quantifying the preservative efficiency of the film, the following measurements were performed: trimethylamine–N and CFU account for the antimicrobial capacity, and peroxide values and fluorescent compound formation for the antioxidant activity. These quantifications permitted the determination that the film successfully extended the shelf-life of the product up to 11 days, maintaining the organoleptic properties [170]. The presence of 8% of algal extracts and 1% of sorbic acid in PLA has been further supported. A work used up to five different genera, *Fucus, Bifurcaria, Gracilaria, Ulva* and *Ascophyllum*, to test their antimicrobial capacity against *B. cereus, B. subtilis, S. aureus, K. pneumoniae, Pseudomonas fluorescens, E. coli, Aeromonas hydrophyla, Vibrio alginolyticus* and *V. parahaemolyticus.* The most effective algae were *Fucus* followed by *Bifurcaria*. Additionally, sorbic acid has been demonstrated to be effective against yeast and molds [171].

Among the molecules present in macroalgae, alginates and fucoidans mostly extracted from brown macroalgae, such as *Sargassum natans*, *S. latifolium* or *L. japonica*, have been repeatedly used for the developing of biodegradable films [172,173,174]. Even though brown algae are the main target for obtaining ingredients for creating films, red algae such as *Gracilaria lemaneiformis* has also demonstrated to possess a protein and polysaccharide composition with promising filming properties [175]. These bio packages have been differently designed using just algae-derived polysaccharides for the development of the film or algae extracts combined with other vegetal molecules such as chitosan [172,173,174,175,176]. The antimicrobial activity of algae polysaccharides can be reinforced with the presence of chitosan since it has been recognized to possess antimicrobial capacity [177]. Furthermore, the phenolic composition of algae extracts contributes to the antioxidant effect. Therefore, films created using algae molecules can be considered themselves as active packaging since they offer a physical barrier while providing biological activities.

Therefore, the use of algae compounds allows the creation of a huge variety of active packaging, which represents an innovative tool for conserving food products. Algae-based active packaging can provide biodegradable films that are free of synthetic molecules showing a positive environmental and economic impact. They would reduce the utilization of single-use plastics and would represent an eco-friendly and safe package that may improve consumers’ acceptance.

### 4.3. Anti-Biofilm and Anti-Fouling

Bacteria have the ability to get attached to solid supports where they form structured communities also known as biofilms [178]. Food can represent a perfect growth media for microorganisms and thus, an easy target for the development of biofilms. Due to the negative economic and health impact that the development of biofilms has for the food industry, new packaging alternatives have been designed. The use of antibiotics has been strongly associated with pathogenic bacteria resistance, which means a global health threat [179]. As aforementioned, macroalgae polysaccharides have been described to possess antibacterial activity [161,178]. However, other compounds present in different algae species have shown antifouling and antibacterial abilities. Polyphenols present in brown algae such as *Fucus*, *Bifurcaria*, *Cystoseira* or *Sargassum* possess antibacterial activity such as fatty acids from red algae genera *Laurencia* and *Chondrus.* Pigments, such as chlorophyll or β-carotene, contained in green algae such as *Ulva* have been described as antibacterial and antifouling [180]. Algae extracts have been considered for their application as antifouling or anti-biofilm coating compounds. As explained above, the incorporation of polysaccharides, such as those extracted from *Fucus* and *Bifurcaria*, into films, such as PLA, provides antimicrobial capacity, which can ultimately avoid biofilm creation [170,171]. Additionally, *Gelidium corneum* active principles were encapsulated into silver nanoparticles, which showed high antimicrobial activity at very low MICs for *Candida albicans* (0.5 μg/mL) and for *E. coli* (0.3 μg/mL). In fact, the antibiofilm efficacy of the 2 μg/mL nanoparticles was demonstrated to be higher than a 70% threat [179].

The potential of macroalgae compounds to prevent biofilm apparition in food matrixes is still under development. Nevertheless, the application of algae extracts in different films, both free and encapsulated, represents a very promising tool for avoiding anti-fouling and bacterial spoilage without requiring the use of harmful antibiotics.

## 5. Food Safety and Quality Control Enhancement Using Antimicrobials from Seaweeds

The main concern for authorities and professionals in the food supply chain is to guarantee food quality and safety [181]. The keeping of the organoleptic characteristics and the nutritional and chemical composition of foods is influenced by the elimination of foodborne microorganisms. Microbial risk is the most important factor in food safety. Microorganisms may affect food safety both through direct ingestion of pathogens present in food and by the ingestion of the microbial toxins excreted in foods by microorganisms during handling before processing [182].Food systems present high susceptibility to microbial contamination, which may diminish their quality properties and therefore, their nutritional value [183].

**Figure 2:**
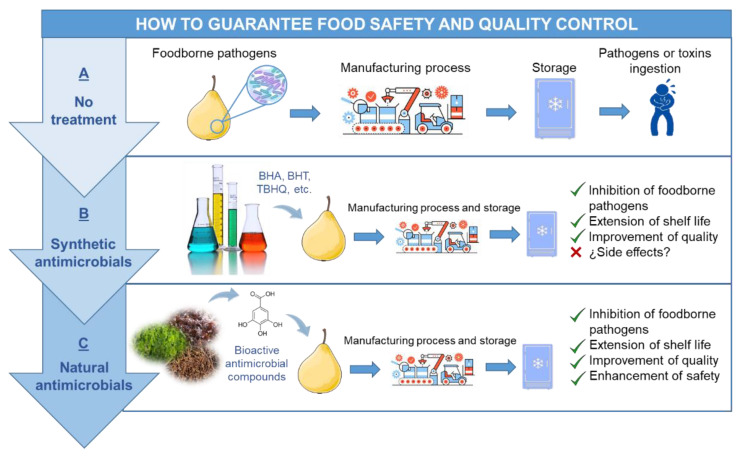
Enhancement of food safety and quality control of fresh and ready-to-eat foods using natural antimicrobials from seaweeds.

Hence, the main problem regarding food safety is foodborne pathogens [184]. Food safety and quality control can be enhanced using antimicrobial agents that can be present naturally or incorporated ad hoc in foods to inhibit microbial growth or cause the death of microorganisms [185]. Foodborne microorganisms are responsible for diseases with a high impact on public health and currently, they are an important concern for the health authorities. In addition to causing infections and intoxications through contaminated foods, pathogenic bacteria are responsible for the food deterioration during storage and distribution with a high impact on food quality, shelf-life and food loss. Therefore, the search for solutions to avoid the deterioration of food caused by pathogenic and spoilage microorganisms has focused the efforts of the food industry, in order to improve food safety and quality [185]. Figure 2 shows an illustration where the general concepts related to food safety and quality control are summarized in a visual form.

Two facts have encouraged the search of antimicrobials in the food industry, on one hand, conventional methods to preserve foods present less efficiency to reduce or inhibit the proliferation of foodborne pathogens and on the other hand, the increased demand for minimally processed foods. The application of antimicrobials is a new strategy to increase the shelf life of food products and overcome the problems of food quality and safety [184]. Preservation of food safety and quality has been extensively carried out by the food industry using synthetic antimicrobials (sodium benzoate, sodium nitrite and sorbic acid) and synthetic antioxidants (butylated hydroxyanisole (BHA), butylated hydroxytoluene (BHT) and tert-butyl hydroxyquinone (TBHQ)). However, in the last few years, it has been reported that these synthetic compounds could have toxic and carcinogenic effects for the consumer [184,186]. Additionally, the interest in antimicrobials from natural sources has also increased due to the incorrect use of antibiotics, which has provoked the development of multidrug-resistant microorganisms, including foodborne pathogens that exhibit resistance to the current antibiotics and are not further affected by the conventional food processing and preservation techniques. From a point of view of food safety and quality control, such natural antimicrobials are the key to solve the drawbacks described above [187]. Nowadays, the increase in consumer demand for minimally processed foods with natural additives and consumer concern about food safety and quality has encouraged the search for alternative natural bio preservatives with antimicrobial properties [184,186].

In this context, in recent years, the use of preservatives from natural sources to avoid or decrease the growth of foodborne microorganisms is a promising approach to replace synthetic ones [184]. Antimicrobial extracts from seaweeds are outlined as alternatives to the synthetic preservatives in the food industry to keep or improve food safety and quality [188]. The possibility of incorporating such extracts in perishable food products, such as refrigerated ready-to-eat foods, could expand their shelf-life, diminish or remove pathogenic bacteria and at the same time, enhance the perception of the consumer to processed food by substituting synthetic with natural antimicrobial agents [189]. Previous sections collect several works about the antimicrobial properties of different seaweeds, which would have potential applications in improving the safety and quality of foods.

## 6. Future Perspectives and Conclusions

Before the creation of modern antibiotics, humanity relied on plants and plant extracts to treat infections and other diseases. Modern antibiotics brought a new age of medicine. Currently, society is facing a major threat to the health of the populations as some bacteria have developed resistance to contemporary antibiotics. This demands a different approach to the problem, perhaps based on materials that were traditionally used but applying modern techniques. Throughout this article, we reviewed the antibacterial effect that some algae extracts can have on several foodborne pathogens responsible for worldwide diseases. There is a clear trend in the current market for the consumption of more eco-friendly and natural products and their incorporation not only in consumers’ diets but also in their personal care and overall health showing a potential market for algae-based medicine [189]. Another concern is that invasive algae are threatening ecosystems. These algae can have an antibacterial effect and be potentially used in treatments, thus reducing the amount of this invasive species and helping to reduce their environmental impact.

Algae and alga extracts are also described as able to improve food quality when they are incorporated in feedstock [190], helping to control infections in fish farms [190]. Nowadays, food safety, functional foods and non-traditional diets are very important issues and algae incorporation in food products is already a reality [191,192]. Therefore, macroalgae are an easily available resource that has great potential as the origin of new active biomolecules capable of positively contributing to the control of foodborne bacteria [193]. This contribution could be used as newfound food preservatives, active packaging or anti-fouling and anti-biofilm agents as discussed previously. Further efforts should be made in the pursuit of appealing organoleptic characteristics as algae and algae compounds could have a very interesting role in food safety in the nearby future.

The present review focused on the possible utilization of algae-based bioactive compounds (such as alkaloids, proteins, polysaccharides, polyphenols, tannins, catechin, fatty acids) [20,72,88] against the proliferation of foodborne pathogens.

There is a considerable amount of data on different algae species and their ability to minimize and even stop bacterial growth. However, reported works show that there are several issues strongly affecting algae extract performance. Extraction conditions and the solvent selection seem to play an important role in the performance of the algae extracts as antimicrobial agents. Nevertheless, even though the pursuit for clean and safe extraction techniques continues, some toxic chemicals such as chloroform, are still used as solvent possibilities. It is also noteworthy that environmental conditions and seaweed harvest location also play a part in the process, being responsible for the variations in the antimicrobial effectiveness of the algae extracts. The demand for natural foods is a worldwide trend, and in that sense, algae are an important resource due to their nutritional properties and particularly due to the bioactive molecules present in their composition. As a result, antimicrobial compounds extracted from algae have shown potential, fundamentally as food preservatives of direct application but also as part of the formulation of active packaging and for other functions such as anti-biofilm or anti-fouling. More applications are expected to be developed. Additionally, algal extracts might contribute towards food safety as they have been proven to be active against foodborne pathogens.

## Figures and Tables

**Table 1:** Antimicrobial effect of algae extracts in *Escherichia coli* (reference strains and serotypes: American Type Culture Collection (ATCC) 8739/10536/11775/25922/35218; MTCC 739; NCIMB 50034; DSM 498; RCM 01002 52-6; PTCC1330, O157:H7; O126:B16).

Seaweed	Solvent	MIC/IC50/MBC (mg/mL)	Agar Diffusion	Ref.
*Laurencia brandenii* (R)	MeOH		Re	[63]
*G. ornata* (R)	H_2_O/EtOH f		NI/NI/R	[64]
*Pterocladia capillacea* (R)	H_2_O _cold/_H_2_0 _hot_		NI/NI	[55]
*Dictyopteris membranacea* (B)			I/NI	
*D. membranacea* (B)	(MeOH:DCM)/MeOH f/AcO f/(MeOH:DCM)		Re/Re/I/NI	[28]
*P. capillacea* (R)/*Osmundaria obtusiloba* (R)	EtOH/Hex		NI	[40]
*Dictyota dichotoma* (B)/*P. pavonica* (B)/*Sargassum vulgare* (B)	AcO	MIC (2.5/2.5/5)		[41]
*Cystoseira amentacea* (B)/*Cystoseira barbata* (B)/*Cystoseira compressa* (B)	AcO	MIC (5)		[46]
*Cystoesira myrica* (B)/*Cystoesira trinodis* (B)/*Padina gymnospora* (B)/*Sargassum dentifolium* (B)/*Sargassum hystrix* (B)/*Actinotrichia fragilis* (R)/*Caulerpa racemosa* (G)/*Codium fragile* (G)	MeOH/EtAc		Re/Re	[47]
*Padina australis* Hauck (B)	MeOH/DCM/n-Hex	MIC (0.88/1.04/1.25)		[48]
*Sargassum polycystum* (B)		MIC (0.88/1.04/1.04)		
*S. polycystum* (B)		MIC (0.73/0.83/0.73)		
*P. australis Hauck* (B)		MIC (2.08/2.08/1.66)		
*S. polycystum* (B)		MIC (0.83/1.04/0.625)		
*S. polycystum* (B)		MIC (0.52/0.83/0.42)		
*Gracilaria* sp. (R)	MeOH		Re	[65]
*L. papillosa* (R)	DCM/(DCM:MeOH)/MeOH/H_2_O	MIC (0.8/0.2/0.8/0.4) MBC (1.4/0.2/1.6/0.6)	Re/Re/Re/Re	[62]
*Padina antillarum* (B)	MeOH/EtAc	MIC (3.75/3.75)	I/I	[66]
*Padina boergeseni* (B)		MIC (7.5/7.5)	I/I	
*Ulva flexuosa* (G)		MIC (1.18/0.93)	S/S	
*Ulva lactuca* (G)/*Enteromorpha intestinales* (G)/*Cladophora vagabunda* (G)	EtOH		R e/Re/I	[67]
*Grateloupia doryphora* (R)	EtOH/MeOH/EtAc		NI	[68]
*Halimeda tuna* (G)/*Melanothamnus afaqhusainii* (R)/*Dictyota indica* (B)/*D. dichotoma var. intricata* (B)/*Sargassum lanceolatum (B)*	EtOH		Re	[57]
*C. trinodis* (B)	DiEt:EtOH:Hex	MIC (4.12)		[49]
*Cladophora glomerata* (G)	EtOH	MIC (>1.25)	I	[50]
*Enteromorpha linza* (G)		MIC (>5)	Re	
*Ulva rigida* (G)		MIC (>10)	Re	
*C. barbata* (B)		MIC (>5)	Re	
*P. pavonica* (B)		MIC (>1.25)	I	
*C. officinalis* (R)		MIC (>1.25)	I	
*Ceramium ciliatum* (R)		MIC (>5)	Re	
*D. membranacea* (B)	H_2_O/EtAc/CHCl_3_		NI	[59]
*Cymodocea nodosa* (G)/*H. tuna* (G)/*C. barbata* (B)/*Codium bursa* (G)	DCM:MeOH		NI	[51]
*Chaetomorpha linum* (G)/*Cladophora rupestris* (G)/*Fucus serratus* (B)/*Halosiphon tomentosus* (B)/*Saccharina latissima* (B)/*Bonnemaisonia hamifera* (R)/*Callithamonion corymbosum* (R)/*Ceramium tenuicorne* (R)/*Dasya baillouviana* (R)/*Delesseria sanguinea* (R)/*Dumontia contorta* (R)/*Polysiphonia elongata* (R)/*Polysiphonia nigra* (R)/*R. confervoides* (R)	DCM		NI	[69]
*G. corticata* (R)	MeOH/DMSO		S/Re	[61]
*G. edulis* (R)	MeOH		Re	
*S. lomentaria* (B)/*P. pavonica* (B)/*Cystoseira mediterranea* (B)/*Hypnea musciformis* (R)/*Spyridia filamentosa* (R)	MeOH		I/I/NI/NI/Re	[54]
*A. fragilis* (R)/*C. myrica* (B)/*Hormophysa cuneiformis* (B)/*L. papillosa* (R)/*Sargassum cinereum* (R)/*Turbinaria turbinata* (B)	MeOH		Re	[52]
*C. barbata* (B)	EtOH		Re	[70]
*Kappaphycus alvarezii* (R)	EtOH/Hot H_2_O		NI	[71]
*Oedogonium* sp. (G)	MeOH/EtOH		NI	[72]
*Stigeoclonium* sp. (G)			I/I	
*Ulothrix* sp. (G)			I/I	
*Nitzschia* sp. (G)			Re/I	
*Bifurcaria bifurcata* (B)	MeOH/DCM		Re/NI	[53]
			Re/Re	
*Hypnea flagelliformis* (R)	MeOH/H_2_O/DCM		NI	[73]
*C. myrica* (B)			NI	
*Sargassum boveanum*(B)			NI	
*Turbinaria conoides* (B)	n-Hex/ClHx/MeOH/(EtOH:H_2_O)		Re/Re/NI	[74]
*Cystoseira tamariscifolia* (B)/*P. pavonica* (B)/*R. confervoides* (R)/*U. lactuca* (G)	MeOH		I/I/I/Re	[60]
*Laurencia obtusa* (R)/*Codium elongatum* (G)/*C. multifida* (B)	AcO	MIC (0.17/2.5/0.16)		[42]
*Fucus vesiculosus* (B)/*Cystoseira baccata* (B)	EtOH	IC50 (2.25/2.5)		[43]
*Saccharina japonica* (B)	CO_2_	MIC (3.4)		[44]
*Enteromorpha prolifera* (G)	PeEt/DiEt/EtAc/MeOH		I/I/R e/I	[58]
*Ulva reticulata* (G)			S/I/Re/I	
*C. myrica* (B)			I	
*P. pavonica* (B)			I	
*Turbinaria triquetra* (B)			I	
*Sargassum portieriatum* (B)			S/I/I/I	
*G. multipartite* (R)			I/I/I/Re	
*Hypnea esperi* (R)/*Caulerpa prolifera* (G)	MeOH		Re/NI	[75]
*Himanthalia elongata* (B)	n-Hex/EtOH/H_2_O	MBC (7.2/6.0/12.5)		[21]
*Sargassum ilicifolium* (B)/*H. cuneiformis* (B)/*S. polycystum* (B)/*Sargassum* sp. (B)/*T. conoides* (B)/*Turbinaria decurrens* (B)/*Turbinaria ornata* (B)	MeOH		Re	[76]
*Sargassum incisifolium*(B)	EtAc		Re	[76]
*L. complanata* (R)	MeOH/Hex f/(Hex:EtAc) f/MeOH f		Re/NI/NI/NI	[77]
*Grateloupia* sp. (R)/*G. corticata* (R)/*Spyridia* sp. (R)/*Meta-mastophora* sp. (R)/*Calloseris* sp. (R)/*Neurymenia fraxinifolia* (R)	MeOH		Re	
*Halymenia* sp. (R)	DiEt		Re	
*U. reticulata* (G)	(MeOH:CHCl_3_:H_2_O)		I	[78]
*U. rigida* (G)	(EtOH:H_2_O)/Hex f/CHCl_3_ f/EtAc f/ButOH f/H_2_O f		Re/NI/I/Re/NI/NI	[45]
			NI/Re/NI/NI/NI	
*U. lactuca* (G)/*D. dichotoma* (B)	MeOH/DiEt/CHCl_3_		Re/Re/S	[79]
			Re/Re/Re	
*U. rigida (G)*	DCM/DCM:MeOH		NI/Re	[80]

**Seaweed information:** (G)—green algae (Chlorophytes); (R)—red algae (Rhodophytes); (B)—brown algae (Phaeophyta); nd—not determined. **Solvents:** AcO—acetone; EtOH—ethanol; MeOH—methanol; DCM—dichloromethane; EtAc—ethylacetate; H_2_O—water; Hex—hexane; CO_2_—carbon dioxide; DMSO—dimethilsulfoxide; CHCl_3_—chloroform; BuOH—butanol; PeEt—petroleum ether; DIEt—diethyl ether; PolySA—polysaccharides agar; f—fraction. **Agar diffusion:** re—resistant ≤ 14 mm I—intermediate 15–19 mm S—susceptible ≥ 20 mm [32]; NI—no inhibition.

**Table 2:** Antimicrobial effect of algae extracts in selected Gram-negative bacteria.

Seaweed	Solvent	MIC/IC50/MBC (mg/mL)	Agar Diffusion	Ref.
***Enterobacter* spp. (*E. cloacae*)**				
*G. ornata* (R)	H_2_O/EtOH f		Re	[64]
*Gracilaria* sp. (R)	MeOH		NI	[65]
*H. cuneiformis* (B)/*S. ilicifolium* (B)/*S. incisifolium* (B)/*S. polycystum* (B)/*Sargassum* sp. (B)/*T. conoides* (B)/*T. decurrens* (B)/*T. ornata* (B)	MeOH		Re	[76]
*L. complanata* (R)*/Grateloupia* sp. (R)*/G. corticata* (R)*/Halymenia* sp. (R)*/Spyridia* sp. (R)*/Meta-mastophora* sp. (R)*/Calloseris* sp. (R)*/N. fraxinifolia* (R)	MeOH		Re	[77]
*U. reticulata* (G)	MeOH:CHCl_3_:H_2_O		S	[78]
***Proteus* spp. (*P. mirabilis*; *P. vulgaris*)**				
*L. papillosa* (R)	DCM/DCM:MeOH/MeOH/H_2_O		Re/Re/Re/Re	
*Sargassum binderi* (B)	H_2_O/EtOH		Re/Re	[99]
*Amphiroa* sp. (R)			Re/Re	
*T. conoides* (B)			Re/Re	
*Halimeda macroloba* (G)			Re/NI	
*Galaxaura rugosa* (R)	EtOH/CHCL_3_ f/n-BuOH f/H_2_O f	MIC (2.5/1.25/2.5/5)	I/S/S/I	[97]
*L. hawaiiana* (R)		MIC (-/2.5/0.625/10)	NI/I/SI	
*Polysiphonia hainanensis* (R)	EtOH/AcO		Re	[29]
*Halidrys siliquosa* (B)	MeOH/Hex;EtAc f	MIC (1.25)		[117]
*T. conoides* (B)	MeOH/CHCl_3_/EtOH/DiEt/PeEt/EtAc/AcO		Re	[101]
*P. gymnospora* (B)			Re	
*S. tenerrimum* (B)			Re/I/Re/Re/I/I/I	
*Padina tetrastromatica* (B)	MeOH		Re	[100]
*Sargassum muticum* (B)	AcO/CHCl_3_/MeOH		Re/Re/Re	[98]
***Salmonella* spp. (S. choleraesuis; S. typhi; S. typhimurium; S. enterica; S. gallinarum)**				
*L. brandenii* (R)	MeOH		Re	[63]
*L. complanata* (R)*/Grateloupia* sp. (R)/*G. corticata* (R)/*Halymenia* sp. (R)/*Spyridia* sp. (R)/*Meta-mastophora* sp. (R)/*Calloseris* sp. (R)/*N. fraxinifolia* (R)	MeOH		Re	[77]
*G. ornata* (R)	H_2_O/EtOH f		Re	[64]
*Cladophora socialis* (G)	AcO/MeOH		S/I	[118]
*Sargassum latifolium* B (B)			I/I	
*Sargassum platycarpum* A (B)			Re/Re	
*D. membranacea* (B)	MeHO:DCM/EtOH f/AcO f		Re/I/Re	[28]
*S. hytrix* (B)	EtAc/MeOH		Re/NI	[47]
*C. racemosa* (G)			Re/NI	
*S. dentifolium* (B)			Re/NI	
*C. myrica* (G)			NI/Re	
*P. gymnospora* (B)			Re/I	
*C. fragile* (G)			Re	
*A. fragilis* (R)			Re/NI	
*C. trinodis* (B)			Re/Re	
*H. tuna* (G)/*D. dichotoma* var intricate (B)/*D. indica* (B)/*M. afaqhusainii* (R)/*S. lanceolatum* (B)	EtOH:H_2_O		Re/I/I/Re/Re	[57]
*C. glomerata* (G)/*E. linza* (G)/*U. rigida* (G)/*C. barbata* (B)/*P. pavonica* (B)/*C. ciliatum* (R)/*C. officinalis* (R)	EtOH		I/Re/I/Re/R e/Re/I	[50]
*D. membranacea* (B)	H_2_O/CHCl_3/_EtOAc		NI/NI/NI	[59]
*S. lomentaria* (B)/*P. pavonica* (B)/*C. mediterranea* (B)/*H. musciformis* (R)/*S. filamentosa* (R)	MeOH		I/NI/NI/NI/NI	[54]
*E. cava* (B)	EtOH/n-Hex/DCM/EtAc/n-BuOH/H_2_O/Ac	MIC (2/NI/NI/0.25/2/NI/9.7 × 10^−4^)		[109]
*C. barbata* (B)	EtOH		Re	[70]
*H. flagelliformis* (R)	MeOH		NI	[73]
*C. myrica* (B)	MeOH			
*S. boveanum* (B)	H_2_O/MeOH/DCM			
*S. japonica* (B)	Raw material/Raw material + catalyst/De-oiled/de-oiled + catalyst		NI/Re/NI/Re	[44]
*H. esperi* (R)/*C. prolifera* (G)	MeOH		NI	[75]
*H. cuneiformis* (B)/*S. ilicifolium* (B)/*S. polycystum* (B)/*Sargassum* sp. (B)/*T. conoides* (B)/*T. ornata* (B)	MeOH		Re	[76]
***Shigella* sp. (reference strains: ATCC 9204 and 1457)**				
*H. cuneiformis* (B)/*S. ilicifolium* (B)/*S. incisifolium* (B)/*S. polycystum* (B)/*Sargassum* sp. (B)/*T. conoides* (B)/*T. decurrens* (B)/*T. ornata* (B)	MeOH:DCM		Re	[76]
*S. muticum* (B)	CHCl_3/_AcO/MeOH		Re	[98]
*C. rubrum* (R)	DiEt/MeOH/EtOH/CHCl_3_		Re/Re/I/Re	[88]
*S. vulgare* (B)			Re/Re/NI/NI	
*S. fusiforme* (B)			Re/NI/Re/Re	
*P. pavonia* (B)			Re/Re/S/Re	
*T. conoides* (B)	MeOH/AcO/PeEt/EtOH/EtAc/CHCl_3_/DiEt		Re	[101]
*P. gymnospora* (B)			I/I/Re/Re/I/R e/I	
*S. tenerrimum* (B)			I/Re/I/Re/I/R e/I	
***Pseudomonas aeruginosa* (reference strains: ATCC 25619/27853/85327/9027; KCTC 1637; DSM 50071; MTCC 2453/424)**				
*L. brandenii* (R)	MeOH		Re	[63]
*G. ornata* (R)	H_2_O/EtOH/EtOH		Re	[64]
*G. changii* (R)	MeOH	MIC (6.25)	Re	[119]
*C. rupestris (G)*	CHCl_3_:MeOH		Re	[120]
*C. trinodis (B)*	(C_2_H_5_)_2_:EtOH:Hex	MIC (6.6)		[49]
*C. myrica* (B)*/C. trinodis* (B)*/P. gymnospora* (B)/*S. dentifolium* (B)/*S. hystrix* (B)/*A. fragilis* (R)/*Caulerpa racemose* (G)/*C. fragile* (G)	MeOH/EtOH		Re	[47]
*P. australis* (B)	MeOH/DCM/n-Hex	MIC (0.26/0.26/0.73)		[48]
*S.polycystum TK* (B)		MIC (0.73/0.21/0.10)		
*S.polycystum CR* (B)		MIC (0.21/0.21/0.10)		
*L. papillosa* (R)	DCM/DCM:MeOH/MeOH/H_2_O		Re/I/Re/Re	[62]
*U. flexuosa* (G)/*P. antillarum* (B)/*P. boergeseni* (B)	EtAc/MeOH		NI	[66]
*G. doryphora* (R)	EtOH/MeOH/EtAc		Re	[68]
*Eisenia bicyclis* (B)	MeOH/Hex/DCM/EtAc/BuOH		NI	[121]
*H. tuna* (G)/*D. dichotoma* var intricate (B)/*D. indica* (B)/*M. afaqhusainii* (R)/*S. lanceolatum* (B)	EtOH/H_2_O		Re	[57]
*C. glomerata* (G)/*E. linza* (G)/*U. rigida* (G)/*C. barbata* (B)/*C. ciliatum* (R)/*C. officinalis* (R)	EtOH		I/Re/Re/eRe/Re/Re	[50]
*D. membranacea* (B)	H_2_O/CHCl_3/_EtAc		NI/NI/NI	[59]
*C. linum* (G)/*C. rupestris* (G)/*F. serratus* (B)/*F. vesiculosus* (B)/*H. tomentosus* (B)/*S. latissima*(B)/*B. hamifera* (R)/*C. corymbosum* (R)/*C. tenuicorn* (R)/*Ceramium virgatum* (R)/*D. baillouviana* (R)/*D. sanguinea* (R)/*D. contorta* (R)/*P. elongata* (R)/*P. nigra* (R)/*R. confervoides* (R)	DCM		NI	[69]
*B. bifurcata* (B)	MeOH/DCM		Re/Re	[53]
*T. conoides* (B)	n-Hex/C_6_H_12/_MeOH/EtOH:H_2_O		NI/R e/Re/NI	[74]
*C. tamariscifolia* (B)/*P. pavonica* (B)/*R. confervoides* (R)/*U. lactuca* (G)	MeOH		I/I/I/Re	[60]
*E. prolifera* (G)	DiEt	MIC (1.25 × 10^−3^)		[58]
*S. portieriatum* (B)	MeOH	MIC (7.5 × 10^−4^)		
*H. esperi* (R)	MeOH		NI	[75]
*C. prolifera* (G)			I	
*H. cuneiformis* (B)/*S. ilicifolium* (B)/*T. ornata* (B)	MeOH		NI/Re/Re	[76]
*H. elongata* (B)	H_2_O/MeOH		Re	[30]
*U. rigida* (G)	EtOH:H_2_O/Hex/CHCl_3_ f/EtOAc f/ButOH f/H_2_O		Re/NI/Re/Re/NI/NI	[45]
*S. vulgare* (B)	DIEt/MeOH/EtOH/CHCl_3_		Re/NI/NI/NI	[88]
*S. fusiforme* (B)			S/Re/Re/NI	
*P. pavonia* (B)			I/NI/I/I	
*C. rubrum* (R)			I/NI/I/NI	

**Seaweed information:** (G)—green algae (Chlorophytes); (R)—red algae (Rhodophytes); (B)—brown algae (Phaeophyta); nd—not determined. **Solvents:** AcO—acetone; EtOH—ethanol; MeOH—methanol; n-BuOH—butanol; DCM—dichloromethane; EtAc—ethylacetate; EtOAc—ethyl ethanoate; H_2_O—water; Hex—hexane; CO_2_—carbon dioxide; DMSO—dimethilsulfoxide; CHCl_3_—chloroform; BuOH—butanol; PeEt—petroleum ether; DIEt—diethyl ether; PolySA—polysaccharides agar; f—fraction. **Agar diffusion:** Re—resistant ≤ 14 mm; I—intermediate 15–19 mm; S—susceptible ≥ 20 mm [32]; NI—no inhibition.

**Table 3:** Antimicrobial effect of alga extracts on *Staphylococcus aureus* (reference strains: ATCC 25923/2940/11632/6538/6462/333591; TISTR517; CMCC(B)26003; BAA-42; PTCC1112; RCM 010027).

Seaweed	Solvent	MIC/IC50/MBC (mg/mL)	Agar Diffusion	Ref.
*C. socialis* (G)	AcO/MeOH		I/S	[118]
*S. latifolium* (B)			I/S	
*S. platycarpum* (B)			Re/I	
*C. elongatum* (G)/*L. obtusa* (R)/*C. multifida* (B)	AcO	MIC (0.63 × 10^3^)		[125]
*H. macroloba* (G)/*S. binderi* (B)/*Amphiroa* sp. (B)/*T. conoides* (B)	AcO		Re	[99]
*U. lactuca* (G)/*C. fragile* (G)/*L. johnstonii* (R)/*Gymnogongrus martinensis* (R)/*D. flabellata* (B)/*Padina concrescens* (B)	AcO:MeOH		Re/NI/I/NI/NI/NI	[127]
*U. flexuosa* (G)/*P. antillarum* (B)/*P. boergeseni* (B)	MeOH/EtAc	MIC (3.75/1.87)	I/S	[66]
		MIC (7.5/3.75)	I/S	
		MIC (15/3.75)	Re/I	
*U. rigida* (G)	DCM		Re	[80]
	DCM:MeOH			
*H. tuna* (G)/*D. dichotoma var intricata* (B)/*D. indica* (B)/*S. lanceolatum* (B)/*D. dichotoma var intricata* (B)/*M. afaqhusainii* (R)	EtOH		Re	[57]
*C. glomerata* (G)/*E. linza* (G)/*U. rigida* (G)/*C. ciliatum* (R)/*C. barbata* (B)/*P. pavonica* (B)/*C. officinalis* (B)	EtOH	MIC (>1.25/>2.5/>2.5/>10/>1.25/>1.25/>5)	I/Re/I/Re/I/I/Re	[50]
*C. nodosa* (G)/*H. tuna* (G)/*C. barbata* (G)/*C. bursa* (G)	MeOH		Re	[51]
*Oedogonium* sp. (G)	MeOH/EtOH		NI	[72]
*Stigeoclonium* sp. (G)			Re/I	
*Ulothrix* sp. (G)			Re	
*Nitzschia* sp. (G)			Re/I	
*E. prolifera* (G)	EtAc	MIC (1.0 × 10^3^)		[81]
*P. pavonica* (B)	PeEt	MIC (1.25 × 10^3^)		
*C. prolifera* (G)/*H. esperi* (R)	MeOH	MIC (0.6/0.5)	I	[75]
*U. reticulata* (G)	H_2_O		S	[128]
*U. rigida* (G)	Hex/CHCl_3_/EtOAc/ButOH/H_2_O/Hex/CHCl_3_/EtOAc/ButOH/H_2_O		NI/NI/Re/Re/Re/I/I/Re/NI/NI	[45]
*Ulva fasciata* (G)/*Chaetomorpha antennina* (G)/*Caula anthus okamurai* (R)/*Ahnfeltiopsis masudai* (R)/*P. hainanensis* (R)/*Sargassum hemiphyllum* (R)/*Sargassum vachellianum* (R)/*Pachydictyon coriaceum* (B)	EtOH		Re/Re/Re/Re/Re/I/I/Re	[29]
*L. brandenii* (R)	CHCl_3_/MeOH		I	[63]
*G. ornata* (R)	MeOH		Re	[64]
*Gracilaria* sp. (R)	PolySA/MeOH		NI	[65]
*L. papillosa* (R)	EtOH/MOH/AcO		Re	[62]
*G. corticata* (R)/*G. edulis* (R)	MeOH/DMSO		Re	[61]
*H. musciformis* (R)/*S. filamentosa* (R)/*S. lomentaria* (B)/*P. pavonica* (B)/*C. mediterranea* (B)	MeOH		Re	[54]
*A. fragilis* (R)/*L. papillosa* (R)/*S. cinereum* (R)/*C. myrica* (B)/*H. cuneiformis* (B)/*T. turbinata* (B)	DMSO		Re	[52]
*H. flagelliformis* (R)	DCM/MeOH		Re	[73]
*C. myrica* (B)			NI	
*S. boveanum* (B)			NI	
*R. confervoides* (R)/*U. lactuca* (G)/*C. tamariscifolia* (B)/*P. pavonica* (B)	MeOH		I	[60]
*L. complanata* (R)*/Grateloupia* sp. (R)*/G. corticata* (R)*/Halymenia* sp. (R)*/Spyridia* sp. (R)*/Metamastophora* sp. (R)*/Calloseris* sp. (R)*/N. fraxinifolia* (R)	MeOH		Re	[77]
*P. gymnospora* (B)	H_2_O	MIC (500 × 10^3^)		[132]
*Sargassum oligocystum* (B)	Hot H_2_O	MIC (3.18)		[129]
*E. bicyclis* (B)	MeOH/Hex/DCM/EtAc/BuOH	MIC (1.02/256/512/128 × 10^3^/512 × 10^3^)	Re	[121]
*C. trinodis* (B)	DIEt:EtOH:Hex	MIC (1.031)		[49]
*S. vulgare* (B)	EtAc		Re	[130]
*D. membranacea* (B)	H_2_O/DCM/EtAc		Re	[59]
*E. cava* (B)	EtOH/n-Hex/DCM/EtAc/n-BuOH/H_2_O	MIC (500 × 10^3^/nd/nd/250 × 10^3^/nd/nd)		[109]
*C. barbata* (B)	EtOH		Re	[70]
*B. bifurcata* (B)	MeOH/DCM		Re/NI	[53]
*D. dichotoma* (B)/*P. pavonia* (B)/*S. vulgare* (B)	AcO	MIC (1.25/1.25/2.5)		[41]
*F. vesiculosus* (B)	EtOH	IC_50_: (1.25)		[43]
*S. japonica* (B)	CO_2_	MIC (3)		[44]
*D. membranacea* (B)	MeOH:DCM/EtOH/AcO		Re/S/S	[28]
*H. elongata* (B)	Hex/EtOH/H_2_O	MBC (8.25/7.00/13.0)		[21]
*H. cuneiformis* (B)*/S. ilicifolium* (B)*/S. incisifolium* (B)*/Sargassum* sp. (B)*/T. conoides* (B)*/T. decurrens* (B)*/T. ornata* (B)	MeOH		Re	[76]
*F. serratus* (B)/*F. vesiculosus* (B)	H_2_O/MeOH/AcO/EtAc		I	[131]

**Seaweed information:** (G)—green algae (Chlorophytes); (R)—red algae (Rhodophytes); (B)—brown algae (Phaeophyta); nd—not determined. **Solvents:** AcO—acetone; EtOH—ethanol; MeOH—methanol; DCM—dichloromethane; EtAc—ethylacetate; H_2_O—water; Hex—hexane; CO_2_—carbon dioxide; DMSO—dimethilsulfoxide; CHCl_3_—chloroform; BuOH—butanol; PeEt—petroleum ether; DIEt—diethyl ether; PolySA—polysaccharides agar; f—fraction. **Agar diffusion:** Re—resistant ≤ 14 mm; I—intermediate 15–19 mm; S—susceptible ≥ 20 mm [32]; NI—no inhibition.

**Table 4:** Antimicrobial effect of alga extracts in selected Gram-positive bacteria.

Seaweed	Solvent	MIC/IC50/MBC (mg/mL)	Agar Diffusion	Ref
***B. cereus* (reference strains: ATCC 10876/11778/13061/14579; CMCC-B-63303)**				
*C. racemosa (G)/C. fragile (G)/A. fragilis (R)/C. myrica (B)/C. trinodis (B)/P. gymnospora (B)/S. dentifolium (B)/S. hystrix (B)*	EtAc		Re	[47]
*C. glomerata (G)/E. linza (G)/U. rigida (G)*	EtAc	MIC (>10)	Re	[50]
*C. officinalis (R)/C. ciliatum (R)/C. barbata (B)/P. pavonica (B)*	EtOH	MIC (>10)	Re	
*P. capillacea (R)/D. membranacea (B)*	Cold H_2_O/Hot H_2_O		Re/NA	[11]
*S. polycystum C, TK (B)*	MeOH/DCM/n-Hex	MIC (0.13/0.21/0.37)		[48]
*S. polycystum C, CR (B)*		MIC (0.21/0.21/0.07)		
*P. australis, CR (B)*		MIC (0.21/0.21/0.07)		
*L. complanata (R)/Grateloupia* sp. *(R)/G. corticata (R)/Halymenia* sp. *(R)/Spyridia* sp. *(R)/Meta-mastophora* sp. *(R)/Calloseris* sp. *(R)/N. fraxinifolia (R)*	MeOH		S/Re/Re/Re/Re/Re/Re/Re	[77]
*S. vulgare* *(B)*	MeOH: CHCl_3_	MIC (3.75) MBC (>15)	Re	[130]
*Cladostephus hirsutus* *(B)*		MIC (1.87) MBC (>15)		
*Rissoella verruculosa (R)*		MIC (7.5) MBC (>15)		
*C. racemosa (G)*	Hex/CHCl_3/_EtAc/EtOH/MeOH/H_2_O	MIC (0.3/0.6/0.3/2.5/1.2/-) MBC (0.3/0.6/0.3/2.5/-/-)		[57]
*Caulerpa sertularioides (G)*		MIC (0.2/0.1/0.2/0.3/1.2/-) MBC (0.6/0.2/0.2/0.6/1.2/-)		
*K. alvarezii (R)*		MIC (1.2/1.2/2.5/-/-) MBC (1.2/1.2/2.5/-/-)		
*K. alvarezi (R)*	EtOH/H_2_O		Re	[71]
*S. ilicifolium (B)/S. incisifolium (B)/H. cuneiformis (B)/S. polycystum (B)/Sargassum* sp. *(B)/T. conoides (B)/T. ornata (B)/T. decurrens (B)*	MeOH		Re	[76]
*C. anthus okamurai (R)/P. hainanensis (R)/Hypnea chordacea (R)/Hypnea japonica (R)/Hypnea boergesenii (R)/L. okamurai (R)/L. chinensis (R)/P. arborescens (B)/1 P. coriaceum (B)/Ulva conglobata (G)/U. fasciata (G)/C. antennina (G)/C. cylindricum (G)*	AcO		Re/Re/Re/Re/Re/Re/Re/Re/I/Re/Re/I/I	[29]
*U. rigida (G)*	MeOH/Hex f/CHCl_3_ f/EtAc f/BuOH f/H_2_O		Re/Re/I/Re/NI/NI	[45]
***Enterococus* spp.: *E. faecalis* (ATCC 29737, 29219); *E. faecium* (ATCC 19434)**				
*C. rupestris (G)*	CHCl_3_: MeOH		Re	[120]
*U. flexuosa (G)*	MeOH/EtAc	MIC (15/3.75)		[57]
*P. antillarum (B)*		MIC (15/7.5)		
*P. boergeseni (B)*		MIC (15/7.5)		
*U. rigida* *(G)*	DCM		Re/I	[80]
	DCM:MeOH		Re/I	
*C. nodosa (G)/H. tuna (G)/C. bursa (G)/C. barbata (B)*	DCM:MeOH		N/D	[51]
*H. musciformis (G)/S. lomentaria (B)/S. filamentosa (R)/P. pavonica (B)/C. mediterranea (B)*	MeOH		ND/Re/ND/Re/ND/ND	[54]
*U. rigida (G)*	Hex f/CHCl_3_ f/EtAc f/BuOH f/H_2_O f		ND/I/Re/ND/ND	[45]
*Grateloupia doryphore (R)*	MeOH/EtAc/EtOH		Re	[68]
*D. membranacea (B)*	MeOH:DCM/EtOH f/AcO f/MeOH/DCM f/MeOH:DCM/EtOH f/AcO f/MeOH:DCM f		I/S/S/Re/Re/Re/Re/Re	[28]
***L. monocytogenes* (reference strains: NCTC 11994; ATCC 19115)**				
*C. glomerata (G)/E. linza (G)/U. rigida (G)/C. officinalis (R)/C. ciliatum (R)/C. barbata (B)/P. pavonica (B)*	EtOH	MBC (>1.25/>2.2/>10/>2.5/>2.5/>2.5/>1.25)	Re	[50]
*U. rigida (G)*	Hex f/CHCl_3_ f		S/Re	[45]
*D. membranacea (B)*	H_2_O		I	[59]
*H. elongata (B)*	H_2_O		Re	[30]
	20% MeOH		Re	
	40% MeOH		Re	
	60% MeOH		Re	
	80% MeOH		Re	
	MeOH		Re	
*C. glomerata (G)/U. rigida (G)/E. linza (G)/C. barbata (B)/P. pavonica (B)/C. ciliatum (R)/C. officinalis (R)*	EtOH		I/Re/Re/Re/Re/Re/Re	[50]
***Streptococcus* spp. (*S. agalactiae*; *S. pneumoniae*; *S. suis*; *S. aureus*; *S. mutans*; *S. pyogenes*)**				
*L. brandenii (R)*	PeEt:DCM (6:4) f		Re	[154]
*C. rupestris (G)*	DCM:MeOH		Re	[120]
*L. papillosa (R)*	DCM/(DCM:MeOH)/MeOH/H_2_O		Re/Re/Re	[155]
*H. cuneiformis, (B)/T. ornata (B)/T. conoides (B)/S. polycystum. (B)/S. ilicifolium (B)/S. incisifolium (B)/T. decurrens (B)/Sargassum* sp. *(B)*	MeOH		Re	[76]
*L. complanata (R)*	MeOH/DCM f/(Pentene:DIEt) f/MeOH f		S/Re/Re/Re	[77]
*G. corticata (R)/Halymenia* sp. *(R)/Spyridia* sp. *(R)/Meta-mastophora* sp. *(R)/Calloseris* sp. *(R)/N. fraxinifolia (R)*	MeOH		Re	[77]
*Ulva armoricana (G)*	SPoly f	MIC (6.25)		[156]
*E. linza (G)*	EtOH/MeOH/AcO		Re/Re/I	[157]
*T. conoides (B)/P. gymnospora (B)/S. tenerrimum (B)*	MeOH/AcO/PeEt/EtOH/EtAc/CHCl_3/_DiEt		Re	[101]
*G. rugosa (R)*	CHCl_3/_n-BuOH/H_2_O		Re/I/S	[97]

*L. hawaiiana (R)*			S/NI/NI	


**Seaweed information:** (G)—green algae (Chlorophytes); (R)—red algae (Rhodophytes); (B)—brown algae (Phaeophyta); nd—not determined. **Solvents:** AcO—acetone; EtOH—ethanol; MeOH—methanol; DCM—dichloromethane; EtAc—ethylacetate; H_2_O—water; Hex—hexane; CO_2_—carbon dioxide; DMSO—dimethilsulfoxide; CHCl_3_—chloroform; BuOH—butanol; PeEt—petroleum ether; DIEt—diethyl Ether; PolySA—polysaccharides agar; f—fraction. **Agar diffusion:** Re—resistant ≤ 14 mm; I—intermediate 15–19 mm; S—susceptible ≥ 20 mm [32]; vs.—very strong; NI—no inhibition.

## Abbreviations

**Generic**	
ATCC	American Type Culture Collection
B	brown algae (Phaeophyta)
BAA	Asian Bacterial Bank
CLSI	Clinical and Laboratory Standards Institute
CFU	Colony Formation Unix
CMCC	China Medical Culture Collection Center
DSM	Deutsche Sammlung von Mikroorganismen
f	fraction
G	green algae (Chlorophyta)
FRAP	Ferric Antioxidant Power
MBC	minimal bactericide concentration
MIC	minimal inhibition concentration
MRSA	methicillin-resistant S. aureus
MTCC	Microbial Type Culture Collection and Gene Bank
NCIMB	National Collection of Industrial, Food and Marine Bacteria
nd	not determined
nt	not tested
NI	no Inhibition
PLA	Polylactic acid
PTCC	Persian Type Culture Collection
GMP	good manufacturing practices
HACCP	Hazard Analysis and Critical Control Point
I	intermediate 15–19 mm
IC50	Half maximal inhibitory concentration
RCM	The Republic Collection of Microogrganisms
Re	resistant ≤ 14 mm
SSOP	sanitation standard operating procedure
R	red algae (Rhodophyta)
S	susceptible ≥ 20 mm
**Compounds**	
ABTS	2,2′-azino-bis(3-ethylbenzothiazoline-6-sulfonic acid)
AcO	acetone
n-BuOH	butanol
ClHx	ciclohexane
CHCl_3_	chloroform
CO_2_	carbon dioxide
DCM	dichloromethane
DMSO	dimethilsulfoxide
DIEt	diethyl ether
DPPH	1,1-diphenyl-2-picryl hydrazyl
EtAc	ethylacetate
EtOAc	ethyl ethanoate
EtOH	ethanol
Hex	hexane
H_2_O	water
MeOH	methanol
n-Hex	n-hexane
PeEt	petroleum ether
PUFA	poly-unsaturated fatty acids
PolySA	polysaccharides agar
